# Novel Preparation and Characterization of Zinc Ricinoleate Through Alkali Catalysis

**DOI:** 10.3390/polym16213016

**Published:** 2024-10-28

**Authors:** Sarah Cohen, Itamar Chajanovsky, Ran Yosef Suckeveriene

**Affiliations:** Department of Water Industry Engineering, Kinneret Academic College on The Sea of Galilee, Zemach 1513200, Israel; sarah.cohen00000@gmail.com (S.C.); itamar@kinneret.ac.il (I.C.)

**Keywords:** zinc oxide, zinc ricinoleate, alkali catalysis, odor removal, pollutant removal

## Abstract

The enhanced thermal and chemical stability of Zinc oxide-based materials make them excellent candidates for the removal of odor-producing pollutants and compounds. The zinc salt of ricinoleic acid, commonly known as zinc ricinoleate, is viewed as the top performer. This article describes an innovative two-step synthesis of zinc ricinoleate, where the first step consists of the preparation of an intermediate compound, methyl ricinoleate, which is synthesized via transesterification of castor oil with methanol and catalyzed by sodium hydroxide. The second step comprises the preparation of zinc ricinoleate through the saponification of methyl ricinoleate in the presence of zinc oxide particles. XRD, FTIR, and NMR spectroscopies confirmed the synthesis of methyl ricinoleate and zinc ricinoleate. HR-SEM and AFM images showed the formation of larger particles, while the thermal stability of the materials was confirmed by TGA.

## 1. Introduction

Zinc ricinoleate (ZR) has attracted significant attention for its ability to remove odorous compounds. The ability of ZR to chemically bond with sulfur and nitrogen-containing compounds makes it one of the prime choices as an indoor-air malodorous substance adsorbent [1]. ZR consists of the zinc salt of ricinoleic acid, i.e., a negatively charged unsaturated 18-carbon fatty acid and Zn^2+^ cations [2,3]. Castor oil, a vegetable oil, is the sole commercial source of ricinoleic acid. Castor oil consists of a natural triglyceride composed of saturated and unsaturated fatty acids in different proportions. Ricinoleic acid accounts for 90% of its total fatty acid constituents [3,4]. The remaining 10% is composed of other unsaturated fatty acids, including linoleic (~4%) and oleic (~3%), and saturated fatty acids such as stearic acid (~1%) [5]. The unique molecular structure of ricinoleic acid, with its hydroxyl group located on the 12th carbon of the backbone chain, enables it to plasticize a large variety of polymers and elastomers [6,7]. To address ecological concerns and meet increasingly stringent environmental regulations, fatty acids derived from bio-renewable vegetable oils may provide a viable alternative as new stabilizer additives [8,9].

Zinc oxide (ZnO)-based nanocomposites are commonly used as pollutant scavengers and adsorbents of organic molecules in wastewater, capitalizing on ZnO’s remarkable properties: it is harmless, stable under harsh environments, and demonstrates excellent photocatalytic activity, rendering it suitable for wastewater treatment [10]. Multiple pollutants are present in industrial wastewater, including dyes, heavy metals, and organic residues [11]. Zafar et al. [12] reported successful azo dye adsorption by zinc oxide nanoparticles prepared via co-precipitation; the highest adsorption capacity was 40 ppm at pH 6. They ascribed the sorption mechanism to the electrostatic attraction between the anionic dye molecules and the positively charged ZnO nanoparticles. ZnO nanorods were also reported to exhibit a high adsorption capacity for As(III), a heavy metal present in aqueous solutions, i.e., 52.63 mg g^−1^ at pH 7 [13]. In another study, polysaccharide-based ZnO nanoparticles, prepared from zinc nitrate at a low calcination temperature, successfully removed considerable copper traces from wastewater [14]. Hassan et al. [15] described the ability of a chitosan/silica/ZnO hybrid nanocomposite to remove up to 293.3 mg g^−1^ of methylene blue from wastewater. Zuliani et al. [16] showed that zinc oxide–castor oil polyurethane hybrids could remove as much as 97% of all volatile organic compounds such as acetic acid.

However, very few studies have been devoted to the synthesis of zinc ricinoleate. It is usually prepared by a one-pot enzyme-catalyzed reaction of castor oil, zinc oxide, and water [17]. However, lipase enzymes are costly, in particular on an industrial scale. Heo et al. [2] reported a catalyst-free electrochemical procedure consisting of the anodization of zinc foil in the presence of ricinoleic acid that achieved up to 95% NH_3_ removal capacity. However, this technique is difficult to scale up further. Zhang et al. [18] prepared zinc salt ricinoleic acid by using a quaternary ammonium salt, namely tetramethylammonium hydroxide pentahydrate, which was first dissolved in methanol and then added to a chloroform solution of acid ricinoleic. The reaction took place under reflux for 30 min. Then, a methanol solution of zinc chloride was added, and the mixture was stirred at room temperature overnight, resulting in a pale yellow solid.

An alternative to enzymes and metal salts is the use of basic catalysts such as NaOH and KOH, which require a two-step reaction: (a) the preparation of an intermediate material, an alkyl ester, the most common of which is methyl ricinoleate (MR) derived from an alkali-catalyzed transesterification of castor oil with methanol [19], and (b) the saponification of methyl ricinoleate with zinc oxide, yielding zinc ricinoleate. The castor oil transesterification results in three molecules of methyl ricinoleate and one molecule of glycerol (byproduct), which is easily eliminated by washing [20,21]. This method potentially provides a novel, cost-effective route to synthesize zinc ricinoleate in the presence of zinc oxide under alkali catalysis, which avoids the use of enzymes and zinc salts, both of which are costly.

To overcome the complexities and cost of these procedures, herein, we describe an innovative preparation for zinc ricinoleate in two steps: (a) transesterification of castor oil with methanol catalyzed by sodium hydroxide (NaOH), resulting in methyl ricinoleate, and (b) saponification of methyl ricinoleate in the presence of different amounts of zinc oxide, yielding zinc ricinoleate. FTIR, NMR, and XRD diffractograms confirmed the synthesis of MR and zinc ricinoleate. HR-SEM and AFM images showed the formation of agglomerated particles, while the thermal stability of the materials was confirmed by TGA. To the best of our knowledge, this is the first time that an alkali-catalyzed synthesis has been used to prepare zinc ricinoleate without the use of enzymes.

## 2. Materials and Methods

### 2.1. Materials

Castor oil (Sigma-Aldrich, Amsterdam, The Netherlands) and methanol (Chen Samuel Chemicals Ltd., Haifa, Israel) were used as reactants without further purification. The zinc oxide nanoparticles were purchased from Sigma-Aldrich (puriss. p.a., ACS reagent, Amsterdam, The Netherlands). Sodium hydroxide (NaOH pellets, >98%), which was used as a catalyst, and 32% Hydrochloric acid were purchased from Daejung Chemicals & Metals Co., Ltd. (Siheung-si, Republic of Korea).

### 2.2. Preparation of Zinc Ricinoleate

**Scheme 1:**
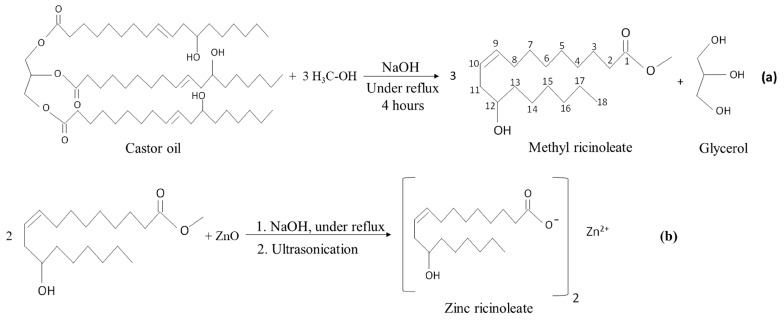
Overall synthesis of zinc ricinoleate (PZR). (**a**) Preparation of the intermediate material, methyl ricinoleate, through the transesterification of castor oil in the presence of methanol. (**b**) Alkali-catalyzed preparation of zinc ricinoleate through the saponification of methyl ricinoleate in the presence of zinc oxide.

The overall route of the two-step reaction for the preparation of zinc ricinoleate is depicted in Scheme 1.

#### 2.2.1. Preparation of Methyl Ricinoleate

An amount of 0.5 g of NaOH pellets (0.5 wt% ratio in castor oil) and a defined amount of methanol corresponding to a molar ratio of methanol:CA 6:1 were added to a 3-necked flask. The mixture was stirred and heated at 50 °C until complete NaOH dissolution. Then, 100 g of preheated castor oil (45 °C) was directly added to the reactor. The transesterification was carried out at 70 °C under reflux for 4 h, resulting in a yellow mixture composed of methyl ricinoleate and the glycerol byproduct [19,21,22]. A schematic figure of the apparatus can be found in the Supporting Information (Figure S1). At high temperatures, glycerol is likely to decompose into acrolein, a very toxic product for human health [19,23]. Therefore, to prevent side reactions in the next stage and acrolein formation, glycerol was extracted from the mixture and analyzed without further purification. The solution was then transferred to a decanter and kept overnight to separate the lower glycerol phase (*d*_glycerol_ = 1.26 g mL^−1^ and *d*_methy ricinoleate_ = 0.923 g mL^−1^). The upper methyl ricinoleate phase was then washed three times with hot water (60 °C) to eliminate all impurities (*d*_hot water_ = 0.983 g mL^−1^). The upper phase containing methyl ricinoleate was then collected (88.7% yield) and used to prepare zinc ricinoleate.

The yield (*r*) was calculated using the following equation: $r=\frac{m_{exp}}{m_{th}}\times 100$, where *m_exp_* (89.1 g) and *m_th_* correspond to the experimental and theoretical mass of MR, respectively. The limiting reactant was castor oil, with 0.1071 mol compared to 0.214 mol methanol. Thus, the MR theoretical mass was equal to $m_{th}=n_{CA}\times 3\times M_{MR}=100.4\;g$ (*M_MR_* is the molecular mass of MR and is equal to 312.49 g mol^−1^).

#### 2.2.2. Preparation of Zinc Ricinolate: Saponification of Methyl Ricinolate

In a 3-necked flask, 0.245 g of NaOH pellets were dissolved in 245 mL distilled water at room temperature (0.5 wt.% NaOH:MR). Then, ZnO nanoparticles were added to the reactor at two different weight ratios of MR:ZnO (1:0.26 and 7:1), followed by the dropwise addition of methyl ricinoleate under continuous stirring, which was then heated to 70 °C under reflux for 12.5 h, for samples PZR1 and PZR2, respectively. The resulting mixture was then sonicated for 1 hour using an ultrasonic liquid processor (750 Watt Sonicator, Sonics & Materials Inc., Newtown, CT, USA) to complete the reaction. The sonication process prevented agglomeration and created small Zn particles. The resulting solution was centrifuged, and the collected product was washed three times with distilled water after centrifugation (Centrifuge 5810 R, Eppendorf AG, Hamburg, Germany; *rpm* = 700; duration cycle = 15 min; room temperature). The resulting white paste-like product was placed in a vacuum oven at 60 °C for 24 h yielding PZR1 (*m*_exp,PZR1_ = 13.51 g, 52.1%) and PZR2 (*m*_exp,PZR2_ = 12 g, 46.3%).

**PZR1**: ^1^H NMR (400 MHzh, CDCl_3_, δ): 0.92 (t, *J* = 6.9 Hz, 3 H; C**H_3_**–CH_2_,), 1.24–1.43 (16 H; alkyl chain, –C**H_2_**–), 1.50 (2 H; –C**H_2_**–CH_3_), 1.65 (2 H; CHOH–C**H_2_**–CH2), 2.02–2.14 and 2.24 (4 H; –C**H_2_**–CH=CH–C**H_2_**–), 2.34 (t, *J* = 6.94 Hz, 2 H; –C**H_2_**–COO^−^), 3.65 (1 H; –C**H**OH), 3.7 (residual MR, 3 H; –COOC**H_3_**), 5.40–5.63 (m, 2 H; –C**H**=C**H**–). ^13^C NMR (400 MHz, CDCl_3_, δ): 14.2 (**C**H_3_–CH_2_), 22.65–36.88 (–**C**H_2_–), 51.49 (residual MR, COO**C**H_3_), 71.53 (**C**HOH), 125.25 and 133.43 (–**C**H=**C**H–), 174.36 (**C**OO^−^).
**PZR2**: ^1^H NMR (400 MHz, CDCl_3_, δ): 0.81 (t, *J* = 6.72 Hz, 3 H; C**H**_3_–CH_2_), 1.14–1.32 (16 H; alkyl chain, –C**H_2_**–), 1.39 (2 H; –C**H_2_**–CH_3_), 1.55 (2 H; CHOH–C**H_2_**–CH2), 1.92–2.03 and 2.14 (4 H; –C**H_2_**–CH=CH–C**H_2_**–), 2.23 (t, *J* = 7.27 Hz, 2 H; –C**H_2_**–COO^−^), 3.55 (1 H; –C**H**OH), 3.6 (residual MR, 3 H; –COOC**H**_3_), 5.22–5.55 (m, 2 H; –C**H**=C**H**–). ^13^C NMR (400 MHz, CDCl_3_, δ): 14.2 (**C**H_3_–CH_2_), 22.65–36.87 (–**C**H_2_–), 51.49 (residual MR, COO**C**H_3_), 71.52 (**C**HOH), 125.26 and 133.38 (–**C**H=**C**H–), 174.37 (**C**OO^−^).

#### 2.2.3. Preparation of Sodium Ricinoleate

An equimolar mixture of methyl ricinoleate and 50% NaOH aqueous solution was added to a 3-necked flask containing 30 mL distilled water. The reaction took place under reflux at 80 °C for 1.5 h. To extract the remaining NaOH, a defined amount of 32% hydrochloric acid was added to the resulting yellow mixture until it reached pH~7. The clear liquid was collected and then centrifuged three times with distilled water, resulting in sodium ricinoleate.

### 2.3. Characterization

Fourier Transform Infrared (FTIR) spectra of the samples were obtained in the range of 400 to 4000 cm^−1^ to determine the chemical composition of the materials collected during the two stages, using a Thermo 6700 FTIR instrument equipped with a Smart iTR diamond ATR device (Thermo Scientific, Waltham, MA, USA).

The ^1^H and ^13^C NMR spectra for the synthesized zinc ricinoleate were recorded on a Bruker Avance III 400 MHz spectrometer (Bruker, Billerica, MA, USA). All samples were dissolved in deuterated chloroform (CDCl_3_).

Powder X-ray diffraction (XRD) patterns of the synthesized zinc ricinoleate were obtained on a Rigaku (MiniFlex 600) diffractometer (Rigaku, Cedar Park, TX, USA) with Cu Kα radiation (*λ* = 1.54 Å) operating at 30 kV and 20 mV. The diffraction data were spread over a 2θ range from 1° to 41°.

The thermal stability of the materials was determined by thermogravimetric analysis (TGA) coupled with MS, using a TA Instruments Q5000 Thermal Gravimetric Analyzer (TA Instruments, New Castle, DE, USA). The temperature ranged from 25 to 600 °C at a heating rate of 10 °C/min, while monitoring for weight loss as a function of temperature. The analysis was conducted in a nitrogen atmosphere at a flow rate of 25 mL min^−1^.

The morphology of zinc ricinoleate was analyzed by High-Resolution Scanning Electron Microscopy (HR-SEM) (Zeiss, Jena, Germany). The samples were sputtered with carbon prior to observation and examined using an HRSEM equipped with a field emission gun (FEG) electron source (Zeiss Ultra-Plus FEG-SEM, Zeiss, Jena, Germany) at an accelerating voltage of 1 keV.

Atomic force microscopic (AFM) measurements were made using an AFM Workshop instrument system (SA-AFM, Hilton Head Island, SC, USA) operated by the noncontact/tapping method of scanning. The probe was a single-beam cantilever with a force constant of ~40 N m^−1^ and a resonance frequency of ~160 kHz. The AFM images were processed using open-source Gwyddion software (Brno, Czech Republic).

## 3. Results and Discussion

### 3.1. Synthesis and Characterization of Methyl Ricinoleate

**Figure 1:**
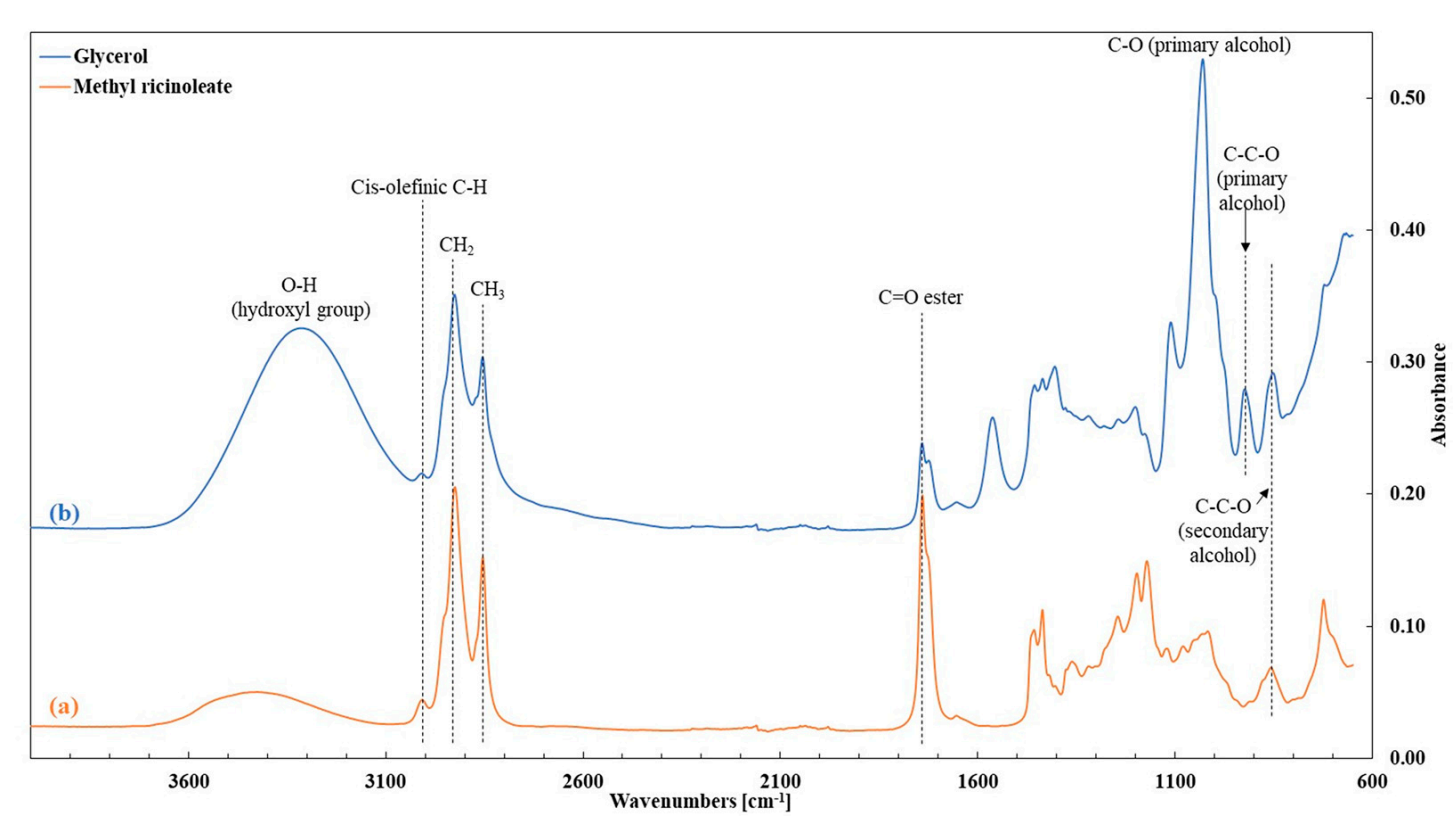
FTIR spectra of (**a**) methyl ricinoleate (orange line) and (**b**) the glycerol byproduct (blue line).

Figure 1 presents the FTIR spectra of methyl ricinoleate (orange line) and the glycerol byproduct (blue line). The preparation of methyl ricinoleate was confirmed by the presence of the main characteristic peaks of the methyl ester group, i.e., at 1740 cm^−1^ and 1171 cm^−1^ (C=O) and 1457–1436 cm^−1^ (C–H of the methyl group). The broad peak at 3426 cm^−1^ corresponds to the hydroxyl group’s stretching vibration located on the 12th carbon of the methyl ricinoleate chain [24,25]. The two peaks at 1121 cm^−1^ and 1080 cm^−1^ correspond to the C–O stretching vibration of the secondary alcohol. The peak at 3007 cm^−1^ corresponds to the *cis*-olefinic C–H stretching vibration of the double bond (C=C) from castor oil [2,26]. The FTIR curve thus confirmed the successful transesterification of castor oil into methyl ricinoleate.

The transesterification of castor oil also produced glycerol, as indicated in Figure 1 by the presence of strong peaks at 3324 cm^−1^ and 1029 cm^−1^, which correspond to the O–H and C–O (primary alcohol) stretching vibration, respectively. The other peaks, in particular at 1741 cm^−1^ and 3009 cm^−1^, correspond to the carbonyl group and the carbon–carbon double bond, respectively, arising from the methyl ricinoleate. The existence of these peaks can be ascribed to either (i) the incomplete separation of glycerol from the methyl ricinoleate, and/or (ii) the presence of impurities, such as other castor oil derivatives, most likely methyl linoleic or stearate [27,28,29]. It is important to note that the transesterification of castor oil always produces a mixture of fatty acid methyl esters, while the largest proportion is methyl ricinoleate, other methyl esters are present in lesser proportions. Note that the obtained glycerol was analyzed without further purification.

### 3.2. Synthesis and Characterization of Zinc Ricinoleate

#### 3.2.1. FTIR Measurements

**Figure 2:**
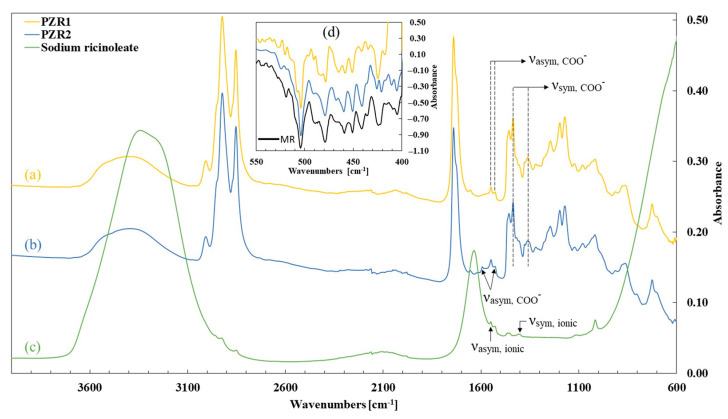
FTIR characterization of the synthesized zinc ricinoleate and sodium ricinoleate. Spectra of (**a**) PZR1 (yellow curve), (**b**) PZR2 (blue curve), (**c**) sodium ricinoleate (green curve), and (**d**) the insert shows the spectra of PZRs and methyl ricinoleate (black curve) in the range 550–400 cm^−1^.

The two PZR products were characterized by FTIR (Figure 2). For both PZRs, in addition to the functional groups due to methyl ricinoleate, two new absorption bands appeared in the region of 1600 to 1500 cm^−1^, indicative of the asymmetric stretching vibration of the carboxylate anion. For PZR1, these peaks were observed around 1548 cm^−1^ and 1526 cm^−1^, whereas for PZR2, they were observed around 1593 cm^−1^ and 1526 cm^−1^. In terms of the symmetric vibration of the carboxylate group, for both PZRs, two peaks were observed around 1435 cm^−1^ and 1362 cm^−1^. The emergence of these new peaks confirms the successful saponification of methyl ricinolate into zinc ricinoleate. The presence of two different peaks in the range of the asymmetric COO^−^ vibration is indicative of the dual coordination mode of the carboxylate group to the metal ion.

The standard way to determine the types of arrangements in carboxylate consists of observing the magnitude of the separation (Δ) between the asymmetric and symmetric peaks [30,31], and then comparing this value to the one calculated from the structural data of the ionic complex, i.e., sodium ricinoleate [18,32]. Note that the vibration frequencies are related to the electron densities of the C–O bond within the carboxylate group [32], so the asymmetric peaks located at 1548 cm^−1^ for PZR1 and 1593 cm^−1^ for PZR2 relate to the symmetric peak at 1362 cm^−1^, with an experimental difference (Δ_1_) equal to 186 cm^−1^ and 231 cm^−1^, respectively. Compared to the Δ value of sodium ricinoleate (Δ_ionic_ = 1555 − 1410 = 145 cm^−1^), ∆_1,PZR1_ was between Δ_ionic_ and 200 cm^−1^, indicating bridging bidentate coordination, whereas Δ_1,PZR2_ was much higher than Δ_ionic_, indicating monodentate carboxylate coordination. Likewise, the peak at 1526 cm^−1^ was observed at 1435 cm^−1^, yielding Δ_2_ = 91 cm^−1^ for both PZRs, which points to the existence of a chelating bidentate arrangement since Δ_2_ was significantly lower than Δ_ionic_ [18,33,34]_._ Therefore, PZR1 presented a combination of both bridging and chelating zinc–carboxylate coordination, whereas PZR2 presented a combination of both chelating and monodentate zinc–carboxylate [30].

As shown in Figure 2, for both PZRs, a new peak appeared in the range of 550–400 cm^−1^, corresponding to metal–oxygen stretching [35]. The band associated with the Zn–O stretching mode appeared at 432 cm^−1^ and 423 cm^−1^ in PZR1 and PZR2, respectively [36]. The shift at higher frequencies for PZR1 indicates a stronger metal–carboxylate bond; it is well-known that bidentate complexes are more stable than monodentate complexes, owing to the formation of a ring with the metal. This change in the crystal structure may be due to the higher volume of ZnO used in PZR1, which resulted in the formation of a supersaturated mixture that led to the difference in crystal nucleation and growth rate [37].

#### 3.2.2. NMR Spectroscopy

**Figure 3:**
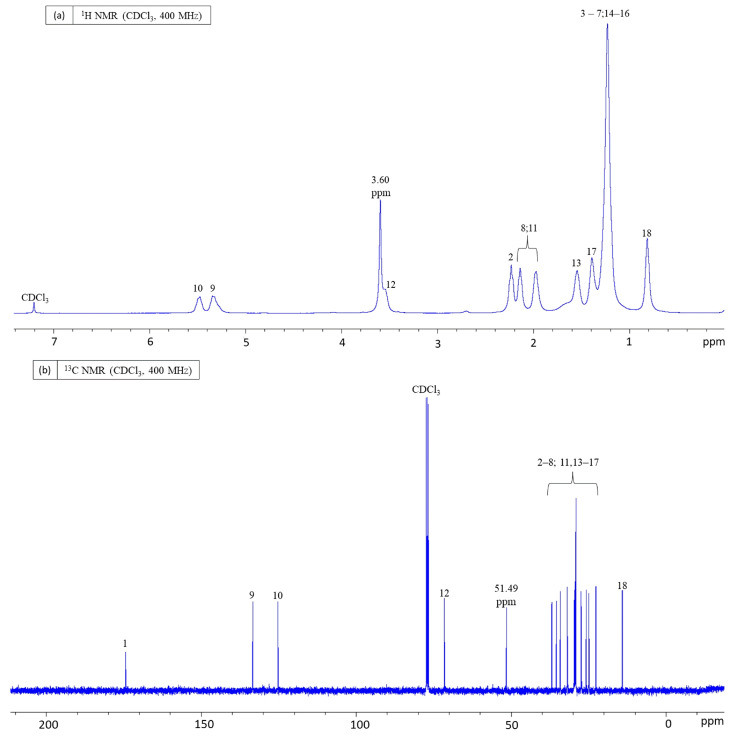
NMR characterization of the synthesized PZR2. (**a**) ^1^H NMR and (**b**) ^13^C NMR.

The synthesized PZRs were then further characterized by NMR. Figure 3 presents the ^1^H NMR and ^13^C NMR spectra for PZR2. PZR1 showed similar spectra. The chemical shifts at 5.22 and 5.55 ppm were ascribed to the two unsaturated protons (–C**H**=C**H**–). The four protons adjacent to the double bond were located at 2.09 and 2.14 ppm (–C**H_2_**–CH=CH–C**H_2_**–). The peak centered at 2.23 ppm corresponds to the methylene protons adjacent to the carboxylate anion (–C**H_2_**–COO^−^). The peak at 3.55 ppm correlates to the single proton attached to the hydroxyl group (–C**H**OH). The peaks in the range of 1.14–1.32 ppm and at 0.81 ppm relate to the remaining methylene groups in the alkyl chain of the fatty acid anion and the terminal methyl protons (CH_2_–C**H_3_**), respectively. However, there is an additional peak at 3.60 ppm, which probably corresponds to methoxy protons from methyl ricinoleate, indicating an incomplete separation between the latter and zinc ricinoleate [29].

In the ^13^C NMR spectrum, the peak at 174.37 ppm corresponds to carbon produced by the carboxylate (–**C**OO^−^), whereas the peaks at 125.26 and 133.38 ppm are attributed to the presence of the carbon double bond (–**C**H=**C**H–). The peak at 71.52 ppm corresponds to the carbon bearing the hydroxyl group (**C**HOH). The peaks in the range of 22.65–36.87 ppm and the peak at 14.2 ppm are ascribed to the remaining methylene carbon (–**C**H_2_–) and the terminal **C**H_3_, respectively. The peak at 51.49 ppm corresponds to the carbon of the methoxy group, thus confirming the presence of methyl ricinoleate residue in PZR2 [4,9,29]. Therefore, the different NMR spectra confirmed the structure of zinc ricinoleate, although the presence of residual methyl ricinoleate was also observed.

#### 3.2.3. X-Ray Diffraction

**Figure 4:**
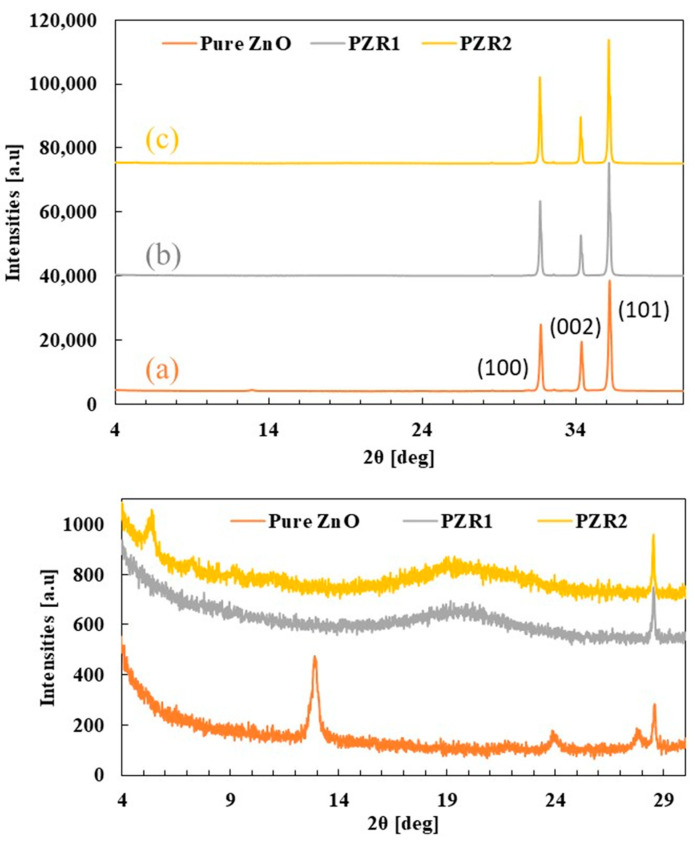
X-ray diffraction characterization of the synthesized PZRs. XDR pattern of (**a**) neat zinc oxide (orange curve), (**b**) PZR1 (grey curve), and (**c**) PZR2 (yellow curve) in the 2θ range of (**Top**) 4–41° and (**Bottom**) 4–30°.

Figure 4 plots the XRD patterns of the neat ZnO and the two synthesized PZRs. All samples showed similar peaks in the 2θ range of 30–38°, centered at approximately 31.72°, 34.38° and 36.21°, corresponding to the Wurtzite ZnO structure (*hkl*) planes (100), (002) and (101), respectively [12,38]. The presence of these characteristic peaks proves the crystallinity of the PZR samples, and therefore confirms the formation of the zinc salt of ricinoleic acid. The diffraction patterns of PZR also show amorphous areas in the 2θ range of 19–23°. The additional diffraction peak observed at 5.32° in PZR2 confirms the differences in zinc ion coordination geometry between PZR1 and PZR2.

In order to determine the structural parameters of the PZR samples, the average crystallite size (*D*) was calculated using the Debye–Scherer equation (Equation (1)) [38,39]:

(1) $$D=\frac{0.9\times \lambda }{\beta \times \cos\theta }$$

where *λ* is the X-ray wavelength equal to 1.5406 Å, *θ* is the Braggs X-ray diffraction angle, and *β* is the full width at half maximum of the peak in radians, according to the (101) peak reflecting the nano-sizes of the PZR samples [40].

The lattice parameters (*a* and *c*), the d-spacing (*d*) and the length of the Zn–O bond (*L*) in the nanostructured PZR samples were calculated using Equations (2)–(5) [38], respectively:

(2) $$a=\frac{\lambda }{\sqrt{3}\sin\theta_{100}}$$

(3) $$c=\frac{\lambda }{\sin\theta_{002}}$$

(4) $$d=\frac{n\lambda }{2\sin\theta },\mathrm{Bragg}\;\mathrm{Equation}$$

(5) $$L=\sqrt{\frac{a^{2}}{3}+{\left(0.5-\mu \right)}^{2}\times c^{2}}$$

where *θ*_100_ and *θ*_002_ are the angles of the peaks (100) and (002), respectively, *n* is the diffraction order equal to 1 for the first order, and *µ* is the positional parameter of the Wurtzite structure equal to $\frac{a^{2}}{3c^{2}}+0.25$.

Table 1 shows that the crystallite sizes of PZR1 and PZR2 was 49.67 nm and 51.05 nm, compared to the neat ZnO, which was 45.29 nm. The length of the Zn–O bond was also affected and was 1.9824 Å and 1.9833 Å for PZR1 and PZR2, respectively, compared to 1.9804 Å for the neat ZnO particles. The smaller crystallite size in PZR1 is the result of using a higher amount of ZnO, which led to a supersaturation; in other words, the nucleation dominated the crystal growth [37]. The PZR lattice parameters are consistent with reports in the literature [41]. The formation of ZnO–carboxylate complexes resulted in new XRD patterns, and the presence of amorphous regions indicates the presence of disordered alkyl chains. Nevertheless, the sharp, high intensity of the diffraction peaks at the higher 2*θ* range in the PZR samples indicate that a certain quantity of ZnO did not react with MR and was not fully eliminated during the washing process, which affected the purity of the samples.

#### 3.2.4. Hr-Sem

**Figure 5:**
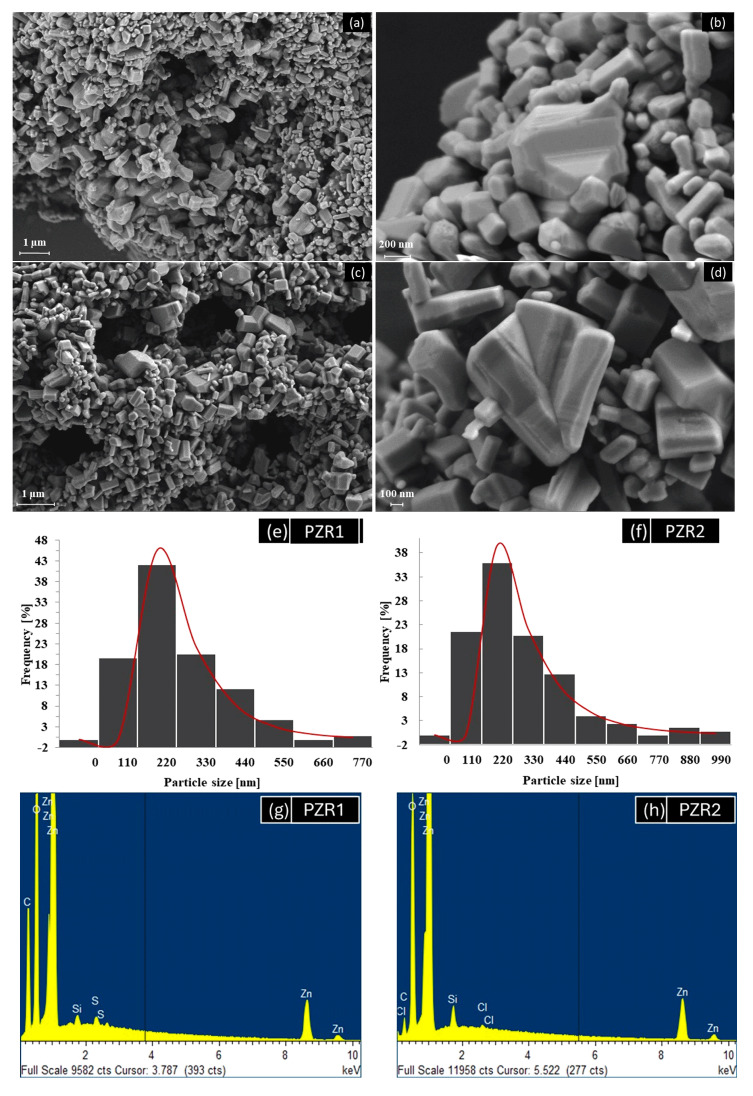
HR-SEM characterization of PZRs. HR-SEM images of (**a**,**b**) PZR1, (**c**,**d**) PZR2. (**e**,**f**) Size distribution and (**g**,**h**) EDS spectra of PZR1 and PZR2.

HR-SEM images of the PZR samples are shown in Figure 5a–d. The HR-SEM images show that the bulk particles had irregular shapes that varied in size from 50 to 990 nm, with an average particle size of 208 nm and 236 nm for PZR1 and PZR2, respectively. The size distribution shown in Figure 5e,f indicates that the smallest particle size was 48.8 nm and 50 nm for PZR1 and PZR2, respectively, which confirms the calculated crystallite size using the Scherrer equation. However, the particle sizes observed in the HR-SEM images were mostly larger than the crystallite size in XRD, thus demonstrating particle agglomeration. The difference in particle size between the two synthesized zinc ricinoleate products can be explained by the higher amount of ZnO used in PZR1, which reduced the alkyl chains’ mobility and, therefore, resulted in smaller particles.

The HR-SEM images did not allow visualization of the fatty acid alkyl chain layer, probably due to the excessive quantity of ZnO used in both preparations. This was confirmed by the Energy Dispersive Analysis (EDS) spectra shown in Figure 5g,h. EDS spectra were taken during the SEM analysis. They revealed that Zn and O were the major constituents, with a weight percentage of elements Zn (L) and O (K) of (17.39–5.09%) and (18.54–4.78%) for PZR1 and PZR2, respectively. The element carbon C (K) was also present at a lower ratio in both samples, confirming the presence of the alkyl chain and, therefore, the successful preparation of zinc ricinoleate. Other elements (Si, S, and Cl) were present in negligible proportions in both EDS spectra. Their presence may have been caused by impurities or contamination during the analysis.

#### 3.2.5. AFM Images

**Figure 6:**
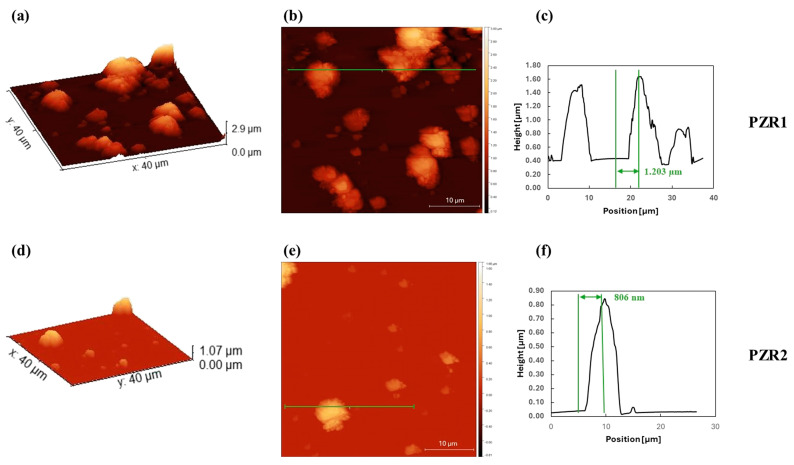
AFM characterization of PZRs. AFM images of (**top**) PZR1 and (**bottom**) PZR2. (**a**,**b**) Surface roughness, (**c**) height profile for PZR1, (**d**,**e**) surface roughness, and (**f**) height profile for PZR2.

Figure 6 shows the surface roughness of the two PZR samples and indicates a thickness on the order of a few micrometers. In addition, the height profiles were examined at a specific cross-section for each sample (green line) and confirmed the presence of both crystalline and amorphous regions in the materials. For PZR1, the height difference was 1.203 µm, and for PZR2, it was 0.806 µm. Interestingly, a greater height and particle agglomeration were observed in PZR1, which seems to contradict the HR-SEM analysis. Although the bulk particle sizes were lower in PZR1, the compactness of this material led to an accumulation of many aggregates, resulting in the formation of a larger agglomeration at the surface.

#### 3.2.6. TGA Measurements

**Figure 7:**
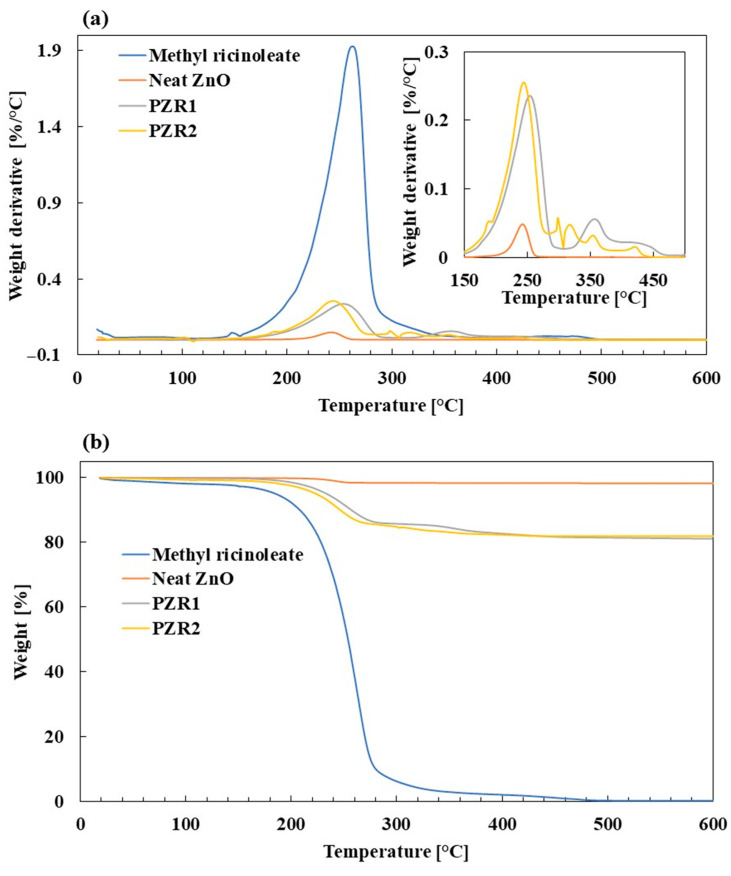
Comparison of the thermal stability of PZRs and MR. (**a**) TGA thermogram of MR (blue curve), neat zinc oxide (orange curve), PZR1 (grey curve), and PZR2 (yellow curve) from 0 to 600°. The insert shows a TGA thermogram in the temperature range of 150–450°. (**b**) DTG thermograms of the same samples.

The thermal stability of the different materials was studied. Figure 7 depicts the TGA and DTG (first derivative) thermograms of neat ZnO particles and synthesized methyl ricinoleate and PZRs. Methyl ricinoleate underwent a two-stage decomposition, with a main transition around 262 °C, which accounts for a weight loss of approximately 96%, followed by a second transition at approximately 455 °C. The first decomposition is due to the degradation of the alkyl chain of fatty acid methyl ester, whereas the small degradation at the higher temperature is due to the thermally stable residue formation [42]. More decomposition steps appeared in the PZRs, showing the formation of a new complex in the presence of ZnO. For PZR1, the first decomposition occurred at around 254 °C, with a weight loss of ~14%, which corresponds to the decomposition of alkyl chains [36]. At approximately 357 °C, the second degradation had a weight loss of ~2.7% due to the decomposition of the carboxylate zinc salt [36]. The final degradation at 433 °C can be considered negligible because the shoulder accounted for a weight loss of less than 1.5% and corresponds to the remaining ZnO. For PZR2, the thermal decomposition process was similar to PZR1, but the degradation peaks occurred at lower temperatures. An additional step was observed between 290 °C and 330 °C. This decomposition is probably related to the presence of carboxylic zinc in the monodentate form.

PZR1 was more thermally stable than PZR2, which can be attributed to its greater chain compactness. This can be attributed to the reduced mobility of alkyl chains in PZR1 that resulted in an increase in van der Waals forces between the chains [9]. The appearance of additional degradation steps at higher temperatures in PZRs compared to MR demonstrates that the presence of ZnO particles in methyl ester resulted in a more thermally stable structure as a result of chelating and bridging bond formation.

## 4. Conclusions

X-ray diffractometry, NMR, FTIR, and TGA measurements confirmed the successful preparation of zinc ricinoleate through a non-enzymatic two-stage procedure under alkali catalysis. Methyl ricinoleate, as an intermediate compound, was first prepared via transesterification of castor oil, and its structure was confirmed by FTIR and TGA. Zinc ricinoleate was then synthesized through the saponification of methyl ricinoleate in the presence of zinc oxide under alkali catalysis. The volume of ZnO particles during this step influenced the zinc ion coordination geometry in the resulting carboxylate–zinc complexes. A combination of both bridging and chelating arrangements was obtained for PZR1, whereas a combination of both chelating and monodentate arrangements was found for PZR2. The incorporation of more ZnO particles caused a supersaturation as well as a reduction in the alkyl chain mobility, which led to the creation of smaller bulk particles. The thermal analysis revealed that PZR1 was more stable than PZR2.

The synthesized zinc ricinoleate materials hold high promise for odor removal. Preliminary panel results indicated a slight decrease in odor. Future experiments and characterizations will further probe these intriguing possibilities.

## Figures and Tables

**Table 1:** Structural parameters of the nanostructured PZRs and neat zinc oxide: crystallite size (*D*), lattice parameters (*a*, *c*), d-spacing (*d*), and Zn–O bond length (*L*) of neat ZnO and PZRs.

Samples	(101) 2*θ*	*D* [nm]	*c* [Å]	*a* [Å]	*c*/*a*	*d* [Å]	*L* [Å]
Neat ZnO	36.2131	45.2944	5.2128	3.2543	1.6018	2.4786	1.9804
PZR1	36.1803	49.6725	5.2177	3.2577	1.6017	2.4807	1.9824
PZR2	36.1658	51.0511	5.2199	3.2591	1.6016	2.4817	1.9833

## Data Availability

The original data presented in this study are openly available in Mendeley Data at 10.17632/dzvr26hr93.1.

## Supplementary Materials

The following supporting information can be downloaded at: https://www.mdpi.com/article/10.3390/polym16213016/s1, Figure S1: Schematic illustration of the synthesis apparatus for (a) Transesterification of castor oil and (b) Liquid–liquid separation (decantation) of the glycerol from methyl ricinoleate.
