# Supplementary material for: Novel Preparation and Characterization of Zinc Ricinoleate Through Alkali Catalysis
Source: Polymers (Basel). 2024 Oct 28;16(21):3016. doi: 10.3390/polym16213016 (PMC11548562; doi:10.3390/polym16213016)
Supplement: Supplementary file 1 [file polymers-16-03016-s001.zip › polymers-3151533-supplementary.pdf]

polymers

Supporting Information

Novel Preparation and Characterization of Zinc Ricinoleat Through Alkali Catalysis

Sarah Cohen, Itamar Chajanovsky and Ran Yosef Suckeveriene *

Department of Water Industry Engineering, Kinneret Academic College on The Sea of Galilee, Zemach, 1513200, Israel; sarah.cohen00000@gmail.com (S.C.); itamar@kinneret.ac.il (I.C.)
* Correspondence: ransots@gmail.com

Preparation of methyl ricinoleate

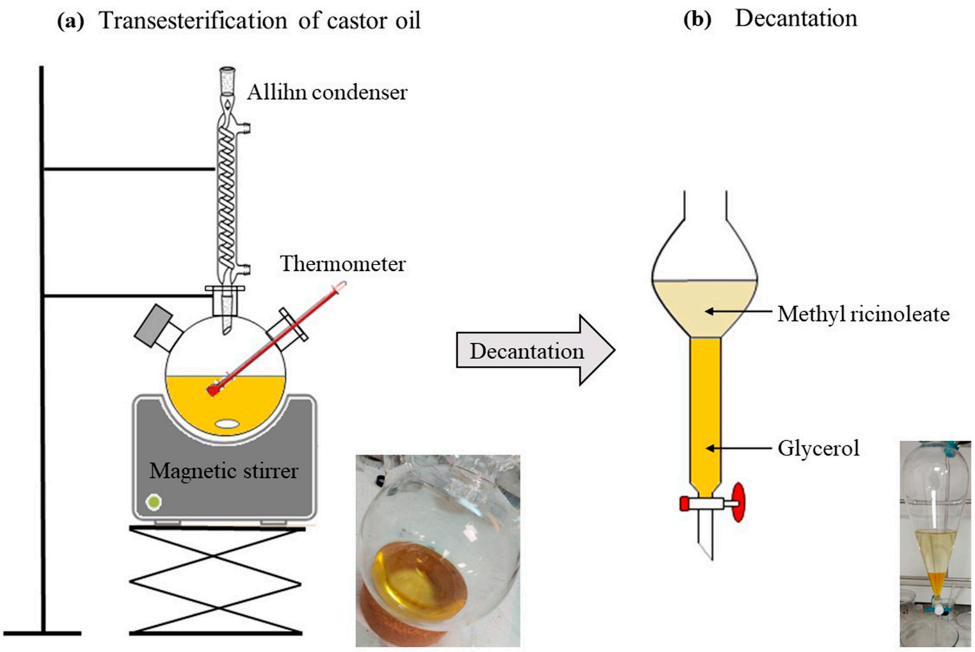

Figure S1. Schematic illustration of the synthesis apparatus for (a) Transesterification of castor oil and (**b**) Liquid-liquid separation (decantation) of glycerol from methyl ricinoleate.
